# The Impact of Screen Time on Sleep Patterns in School-Aged Children: A Cross-Sectional Analysis

**DOI:** 10.7759/cureus.55229

**Published:** 2024-02-29

**Authors:** Chandra Sekhar G, Haarika V, Kedarnath Reddy Tumati, Uma Mahesh Ramisetty

**Affiliations:** 1 Department of Paediatrics, Narayana Medical College and Hospital, Nellore, IND

**Keywords:** social interactions, parental involvement, children, circadian rhythm, physical activity, academic performance, dream recall, sleep patterns, screen time

## Abstract

Background: In a world filled with technology, there's concern about the impact of screen time on children and teenagers. A recent study of 11,875 children aged nine to 10 in the US explored how screen time affects mental health, behaviour, school performance, sleep, and friendships. The results indicate that more screen time is mildly linked to worse mental health, more behaviour problems, lower academic performance, and poorer sleep, but slightly better peer relationships. However, these connections were weak, with socioeconomic status (SES) having a stronger influence on these outcomes. The study doesn't prove that screen time causes these issues, suggesting that increased screen time might not be directly harmful to children in this age group. The research adds to the understanding of how screen time impacts children's well-being and highlights the importance of promoting healthy habits. Our study aims to add to this literature by examining how screen time impacts sleep quality, dream recall, and academic performance in school-aged children. Understanding the potential consequences of screen time is crucial for promoting healthy habits and ensuring the overall well-being of children. Our research seeks to provide further insights into the relationship between screen time and key aspects of children's lives as its prevalence continues to rise.

Methods: The research incorporates a cohort of 1000 school-aged children, bifurcated evenly into two categories based on their daily screen exposure: a low screen time group (less than one hour daily, n = 500) and a high screen time group (over three hours daily, n = 500). The study probes into the correlation between screen time and various health parameters, such as sleep patterns, dream recall, and academic prowess.

Results: The analysis showed that children with low screen time had better sleep efficiency (90% vs. 75%), more frequent dream recall (70% vs. 30%), fewer nocturnal awakenings (0.5 vs. 1.5 times weekly), less daytime sleepiness (20% vs. 60%), and higher physical activity levels (60 vs. 30 minutes per day) compared to those with high screen time. They also had less weekend sleep variability (1.2 hours) and a lower risk of circadian rhythm disturbances (20% increased risk in the high screen time group). Additionally, a screen-free bedroom was more common in the low screen time group (85% vs. 30%), and parental involvement in sleep routines was higher (90% vs. 40%). Academically, the low screen time group achieved higher grades ('A' to 'A+' vs. 'B'), and they had more face-to-face social interactions (two vs. one hour per day).

Conclusion: Escalating screen time is correlated with detrimental impacts on sleep efficiency, dream recall, physical activity, circadian rhythms, and academic performance in school-aged children. Fostering a screen-free bedroom environment and augmenting parental involvement in sleep routines may alleviate these adverse effects.

## Introduction

Digital technology has become a major part of our lives, and children are using screens like smartphones, tablets, computers, and TVs more than ever [1]. While these technologies have many benefits, there are concerns about how too much screen time might affect children's health, especially their sleep and how well they do in school [2]. A recent study of 11,875 children aged nine to 10 in the US explored how screen time affects mental health, behaviour, school performance, sleep, and friendships. The results indicate that more screen time is mildly linked to worse mental health, more behaviour problems, lower academic performance, and poorer sleep, but slightly better peer relationships. However, these connections were weak, with socioeconomic status (SES) having a stronger influence on these outcomes. The study doesn't prove that screen time causes these issues, suggesting that increased screen time might not be directly harmful to children of this age group. The research adds to the understanding of how screen time impacts children's well-being and highlights the importance of promoting healthy habits [1].

Sleep is very important for children's growth and learning. It helps with memory, emotions, and overall health. Experts recommend certain amounts of sleep for different ages to help children grow and develop well [3,4]. But using electronic devices, especially before bed, might make it harder for children to sleep well [5,6].

The objective of this study is to explore into the intricate relationship between screen time and various facets of children's well-being, focusing on sleep patterns, dream recall, and academic performance. Recognising the need for evidence-based insights in this evolving digital landscape, we sought to investigate how the duration and nature of screen exposure may influence these crucial aspects of children's lives.

Sleep and dreams

Sleep has different stages, each important for thinking and feeling. Too much screen time might make it harder for children to sleep well and could change how often they remember their dreams. Our study adds to what we know by looking at how screen time affects children's sleep and dream recall [7,8].

School performance

We're also interested in how screen time affects how children do in school. As schools use more digital tools, it's important to know if screen time changes how children think, pay attention, and do in their classes. Previous studies have had mixed results, so we want to learn more. Our study looks at how children's grades might be linked to how much time they spend on screens [9,10].

Research gap and reason for study

There's a lot of research on how screen time affects some parts of children's health, but we need to know more about its impact on sleep, dreams, and school performance [11,12]. Our study looks at both how much time children spend on screens and how it affects them. We hope our findings will help parents, teachers, and policymakers make better rules and plans to help children use screens in a healthy way.

## Materials and methods

Study design

This prospective cross-sectional cohort study aims to meticulously observe and analyze the impact of screen time (low and high) on sleep patterns, dream recall, and academic performance in children over a period of 12-14 months. 

Low Screen Time

Participants falling into this category reported less than two hours of screen time per day. This threshold was informed by existing literature suggesting that up to two hours of screen time does not significantly interfere with sleep patterns for the demographic in our study [1-5].

High Screen Time

This category was assigned to participants who reported more than four hours of screen time per day. This is based on research indicating that more than four hours can disrupt sleep and cognitive function [1-6].

We established these categories after a thorough review of the literature and current guidelines regarding screen time and its impact on health. Our thresholds are also aligned with recommendations from health authorities and previous empirical studies examining the effects of screen time on sleep and general well-being [1-8].

In this study, we included 1000 participants and divided them into two groups (low and high screen time). The participants were stratified based on key demographic variables relevant to our research question, such as age, gender, and socioeconomic status [1-4]. Random sampling was then employed within each stratum to select individuals, resulting in a sample that reflects the broader population's composition.

The process ensured that every individual within the initial pool had an equal chance of being selected, and the stratification helped maintain the representativeness of our sample in terms of the variables of interest. We believe this method provides us with a robust and unbiased sample for our research objectives.

Participants

Children aged six to 14, enrolled in local schools affiliated with Narayana Medical College in Nellore, form the study group. This age range is a critical developmental stage with significant changes in physical, cognitive, and sleep patterns. Children in this group are increasingly exposed to screens for education, entertainment, and socialization. Understanding how screen time affects their sleep, academic performance, and overall development is crucial. Habits formed during this period can last into later life, so studying this age group can help develop interventions to promote healthy screen habits and sleep hygiene. Additionally, the findings can inform policies and guidelines to support the well-being of school-aged children. Parental or legal guardian consent and child assent are prerequisites for participation.

Inclusion and exclusion criteria

Inclusion Criteria

Children aged between six to 14 who are enrolled in a school participating in the programme are eligible. Additionally, it is essential that there is consent and readiness from both parents/guardians and the children themselves to actively participate and adhere to the study's requirements.

Exclusion Criteria

Children who have been diagnosed with sleep disorders or are on medication that affects sleep, as well as those with cognitive or developmental disorders that impact their ability to understand or report screen time, are excluded from the study. Additionally, children with visual impairments that significantly limit their use of screens are not eligible. The assessment of screen time in this study will be conducted using a dual approach. Firstly, through self-reported data, parents or guardians will record the daily screen usage of their children, including the type and content of the screens used.

Objective Measures

The objective is to accurately track screen exposure by using wearable devices to measure various factors and outcomes.

Sleep Patterns Assessment

The assessment was conducted using wearable sleep monitors, such as sleep tracking bands or rings, which tracked various sleep parameters including sleep stages, heart rate, and movement during sleep [1-4]. Based on these sleep efficiency has been calculated.

Sleep Diaries

Individuals kept a sleep diary to record their sleep and wake times, sleep quality, duration of sleep, and any sleep disturbances. Sleep diaries were often used in conjunction with other assessment methods to provide subjective data on sleep patterns [2-5].

Actigraphy

These devices, worn on the wrist, used accelerometers to track movement and sleep-wake patterns, providing data on sleep duration, quality, and disturbances [2-4].

Dream Recall Assessment

This was done using dream diaries, where participants were asked to record their dreams in detail immediately upon waking, noting the content, emotions, and sensory experiences of the dream. Morning interviews were also conducted, where participants were interviewed upon waking, either in person or via phone, to discuss and record their dreams, allowing for the collection of detailed dream reports. Additionally, structured questionnaires, such as the Dream Recall Frequency Scale or the Mannheim Dream Questionnaire, were used to assess the frequency and nature of dream recall. These questionnaires were adapted to suit the cultural context of India, ensuring that the assessment was relevant and meaningful to the participants [1-3,6-8].

Based on these assessments sleep efficiency has been calculated.

Data analysis

The study employs a range of statistical analyses, including descriptive statistics to outline the sample characteristics, correlation analyses to explore relationships between screen time and health parameters, independent samples t-tests and chi-square tests for group comparisons, logistic regression for assessing the likelihood of circadian rhythm disturbances or specific academic grades, ANOVA for potential multi-group comparisons, and various frequency analyses to present percentages and associations between screen time and sleep-related, physical activity, and academic performance factors.

Ethical considerations

This study was approved by the Institutional Ethics Committee (reference number IEC/NMC/2-23-0078) at Narayana Medical College in Nellore, Andhra Pradesh, India. It makes sure that the participants' information and the data collected are kept private and secure at all times.

## Results

Among the 1000 participants, the low screen time group (n = 500) demonstrated significantly better outcomes in sleep efficiency, dream recall frequency, and academic performance compared to the high screen time group (n = 500). Specifically, children with low screen time showed a sleep efficiency of 90%, a dream recall frequency of 70%, and were more likely to be in the 'A' to 'A+' range in the Andhra Pradesh grading system. In contrast, the high screen time group exhibited a sleep efficiency of 75%, a dream recall frequency of 30%, and generally fell into the 'B' grade category.

Impact of screen time on sleep patterns and dream recall

The study assessed the impact of screen time on sleep patterns and dream recall among children aged six to 14. Participants were divided into two groups based on their daily screen time: low (less than one hour) and high (more than three hours) (Table 1).

**Table 1:** Sleep patterns and dream recall Low: Low screen time; High: High screen time group; P value<0.01 for Efficiency, Dream Recall Frequency, Night Wakings (per week), and Daytime Sleepiness (%), are derived from independent samples t-tests for Sleep Efficiency, Night Wakings, and Daytime Sleepiness, while a chi-square test or Fisher's exact test have been used for Dream Recall Frequency, all indicating statistically significant differences (p < 0.01) between the two groups.

Variables	Low Screen Time Level (N, 500)	High Screen Time Level (N, 500)
Sleep Efficiency (Mean ± SD)	90% ± 5%	75% ± 5%
Dream Recall Frequency (Mean ± SD)	70% (at least once a week) ± 15%	30% (regular dream recall) ± 10%
Night Wakings (per week) (Mean ± SD)	0.5 ± 0.2	1.5 ± 0.5
Daytime Sleepiness (%) (Mean ± SD)	20% ± 10%	60% ± 20%
P-Value (Mean ± SD)	<0.01	<0.01

Sleep efficiency

Children in the low screen time group demonstrated significantly higher sleep efficiency (90%) compared to the high screen time group (75%), with a p-value of <0.01. This suggests that increased screen time is associated with decreased sleep efficiency.

Dream recall frequency

Children with low screen time reported a higher frequency of dream recall, with 70% recalling dreams at least once a week. In contrast, only 30% of children with high screen time reported regular dream recall. This difference was statistically significant, with a p-value of <0.01, indicating a potential link between screen time and dream recall patterns.

Night waking and daytime sleepiness

The low screen time group experienced fewer night wakings (0.5 per week) and lower daytime sleepiness (20%) compared to the High screen time group, which reported 1.5 night wakings per week and 60% daytime sleepiness. These differences were statistically significant, with p-values of <0.01.

Physical health and lifestyle outcomes

Physical Activity

Children with low screen time engaged in an average of 60 minutes of physical activity per day, while those with high screen time engaged in only 30 minutes per day. This difference was statistically significant, with a p-value of <0.05, highlighting a potential negative association between increased screen time and physical activity levels.

Weekend Sleep Variability and Circadian Rhythm Disruption

The high screen time group exhibited higher variability in total sleep duration during weekends (1.2 hours) compared to the low screen time group (0.8 hours). Additionally, children with high screen time showed a 20% higher likelihood of circadian rhythm disruption compared to those with low screen time. Both of these differences were statistically significant, with p-values of <0.05 (Table 2).

**Table 2:** Physical health and lifestyle factors Low: Low screen time; High: High screen time group; Independent samples t-tests for Physical Activity and Weekend Sleep Variability, and a chi-square or Fisher's exact test for Circadian Rhythm Disruption for p<0.05

Variables	Low Screen Time Level (N, 500)	High Screen Time Level (N, 500)
Physical Activity (minutes per day) (Mean ± SD)	60 ± 15	30 ± 10
Weekend Sleep Variability (hours) (Mean ± SD)	0.8 ± 0.3	1.2 ± 0.4
Circadian Rhythm Disruption (%) (Mean ± SD)	5% ± 2%	20% ± 5%
P-Value	<0.05	<0.05

Environmental factors and behavioral habits

Screen-Free Bedroom Environment

A significant difference was observed in the prevalence of a screen-free bedroom environment. While 85% of children with low screen time had a screen-free bedroom, only 30% of children with high screen time maintained this practice. This difference was highly significant, with a p-value of <0.001.

Parental Involvement in Sleep Routines

Children with low screen time reported higher levels of parental involvement in sleep routines, with 90% having a consistent bedtime routine involving their parents. In contrast, only 40% of children with high screen time reported such involvement. This difference was also highly significant, with a p-value of <0.001 (Table 3).

**Table 3:** Environmental factors and behavioral habits Low: Low screen time; High: High screen time group; P<0.01, Chi-square test

Variables	Low Screen Time Level (N, 500)	High Screen Time Level (N, 500)
Screen-Free Bedroom Environment (%) (Mean ± SD)	85% ± 10%	30% ± 15%
Parental Involvement in Sleep Routines (%) (Mean ± SD)	90% ± 5%	40% ± 20%
P-Value	<0.001	<0.001

Academic performance and social interaction

Academic Performance

In this study, children with low screen time demonstrated notably better academic performance. These children achieved an average grade point in the range of 'A' to 'A+', indicative of high academic proficiency. On the other hand, children who engaged in high screen time typically secured grades in the 'B' range, reflecting a comparatively lower academic achievement. This variance in academic performance, aligning with the grading system in Andhra Pradesh, was found to be statistically significant, with a p-value of <0.05.

Social Interaction

A notable difference in face-to-face social interactions was observed between the two groups. Children with low screen time spent an average of two hours per day in face-to-face social interactions, whereas those with high screen time spent only one hour per day in such interactions. This difference was statistically significant, with a p-value of <0.05 (Table 4).

**Table 4:** Academic performance and social interaction Low: Low screen time; High: High screen time group; P<0.05, ANOVA test

Variables	Low Screen Time Level (N, 500)	High Screen Time Level (N, 500)
Average Grade Point (Mean ± SD)	'A' to 'A+’ (Mean Grade: A±)	'B’ (Mean Grade: B±)
Weekly Social Media Usage (hours) (Mean ± SD)	1.5 ± 0.5	5.0 ± 1.0
Extracurricular Involvement (hours per week) (Mean ± SD)	5 ± 1	2 ± 0.5
P-Value	<0.05	<0.05

## Discussion

Our study at Narayana Medical College in Nellore contributes to the burgeoning field exploring the intricate relationship between screen time and various dimensions of children's well-being. The following discussion delves into the implications of our findings on sleep patterns, dream recall, academic performance, and the influence of environmental factors and behavioural habits.

The negative association between screen time and sleep outcomes has been extensively documented. Hale and Guan (2015) highlight that screen time before bedtime, such as watching TV or using the internet, leads to shorter and delayed sleep in school-aged children and adolescents. The blue light that screens emit interferes with the body's circadian rhythm and the production of melatonin, a hormone that is essential for sleep regulation, which may be the cause of this sleep disruption [13]. Additionally, the stimulating content often consumed through screens may also contribute to heightened alertness, further delaying sleep onset. The improved sleep quality observed with reduced screen time may enhance the ability to enter REM sleep, where dreaming primarily occurs, hence the increased dream recall [14].

The impact of screen time on academic performance, as detailed by Pérez-Chada et al. (2023), is multifaceted. The disrupted sleep patterns caused by excessive screen usage can lead to increased daytime sleepiness, negatively affecting children's attention span, memory, and learning capabilities in school. The cognitive overload from constant screen interaction may also contribute to reduced academic performance, as it can impair children's ability to process and retain new information effectively. Thus, managing screen time is essential not only for health but also for educational success [15].

Domingues-Montanari (2017) emphasizes the role of environmental and behavioural factors in promoting healthy sleep patterns. A screen-free bedroom environment minimizes distractions and stimuli that can delay sleep onset. Additionally, involving parents in setting and maintaining sleep routines helps establish a consistent sleep-wake schedule, which is essential for quality sleep. Educating families about the importance of sleep hygiene, including limiting screen time before bed, ensuring a quiet and dark sleep environment, and maintaining regular sleep times, can significantly benefit children's sleep health [16].

The pandemic has resulted in increased reliance on digital devices for education and entertainment, as noted by Zhao et al. (2018). This surge in screen time has been detrimental to children's sleep patterns, with many experiencing reduced sleep duration and quality. The unprecedented shift to online learning and limited outdoor activities have contributed to this issue. Addressing these sleep disturbances is critical, particularly during such challenging times. Strategies could include setting strict limits on screen time, especially before bedtime, encouraging physical activity, and maintaining a routine to help children adapt to the new normal while ensuring adequate rest [17].

The findings of this study have important clinical implications for healthcare professionals, educators, and parents. Clinicians should actively inquire about screen time habits during routine pediatric assessments, considering its potential impact on sleep and academic performance. Educational interventions focusing on promoting healthy screen habits and fostering a screen-free sleep environment may contribute to improved sleep outcomes in children.

Limitations and future directions

While this study provides valuable insights, certain limitations should be acknowledged. The reliance on self-reported screen time and sleep data introduces the possibility of recall bias. Future research could incorporate more objective measures, such as continuous monitoring of screen exposure and sleep parameters. Additionally, longitudinal studies could explore the long-term impact of screen time on developmental trajectories.

## Conclusions

Our research illuminates the complex effects of screen time on school-aged children, revealing connections to their sleep quality, frequency of dream recall, and academic achievements. These insights emphasize the need for a measured and conscious approach to screen use, recognising its potential impact on different facets of a child's life. Understanding and minimizing the effects of screen time on children's well-being is crucial in a world where digital interactions predominate more and more. Such efforts are key to fostering their all-around development and securing the well-being of future generations.

## References

[1] Paulich KN, Ross JM, Lessem JM, Hewitt JK (2021). Screen time and early adolescent mental health, academic, and social outcomes in 9- and 10- year old children: Utilizing the Adolescent Brain Cognitive Development ℠ (ABCD) Study. PLoS One.

[2] Murugan A (2019). Pattern of screen time among middle school students in Chennai, Tamil Nadu. Int J Prev Curat Comm Med.

[3] Akbayin M, Mulliez A, Fortin F, Vicard Olagne M, Laporte C, Vorilhon P (2023). Screen exposure time of children under 6 years old: a French cross-sectional survey in general practices in the Auvergne-Rhône-Alpes region. BMC Prim Care.

[4] Mortazavi S, Motlagh M, Qorbani M (2019). Association of screen time with sleep duration in school-aged children; a nationwide propensity score matched analysis: the Caspian V study. J Res Health Sci.

[5] Kubiszewski V, Fontaine R, Rusch E, Hazouard E (2014). Association between electronic media use and sleep habits: an eight-day follow-up study. Int J Adolesc Youth.

[6] Baiden P, Tadeo SK, Peters KE (2019). The association between excessive screen-time behaviors and insufficient sleep among adolescents: findings from the 2017 youth risk behavior surveillance system. Psychiatry Res.

[7] Hiltunen P, Leppänen MH, Ray C (2021). Relationship between screen time and sleep among Finnish preschool children: results from the DAGIS study. Sleep Med.

[8] Parent J, Sanders W, Forehand R (2016). Youth screen time and behavioral health problems: the role of sleep duration and disturbances. J Dev Behav Pediatr.

[9] Yland J, Guan S, Emanuele E, Hale L (2015). Interactive vs passive screen time and nighttime sleep duration among school-aged children. Sleep Health.

[10] Bapat R, van Geel M, Vedder P (2017). Socio-economic status, time spending, and sleep duration in Indian children and adolescents. J Child Fam Stud.

[11] Alshoaibi Y, Bafil W, Rahim M (2023). The effect of screen use on sleep quality among adolescents in Riyadh, Saudi Arabia. J Family Med Prim Care.

[12] Maurya C, Muhammad T, Maurya P, Dhillon P (2022). The association of smartphone screen time with sleep problems among adolescents and young adults: cross-sectional findings from India. BMC Public Health.

[13] Hale L, Guan S (2015). Screen time and sleep among school-aged children and adolescents: a systematic literature review. Sleep Med Rev.

[14] Oka Y, Suzuki S, Inoue Y (2008). Bedtime activities, sleep environment, and sleep/wake patterns of Japanese elementary school children. Behav Sleep Med.

[15] Pérez-Chada D, Bioch SA, Schönfeld D, Gozal D, Perez-Lloret S (2023). Screen use, sleep duration, daytime somnolence, and academic failure in school-aged adolescents. PLoS One.

[16] Domingues-Montanari S (2017). Clinical and psychological effects of excessive screen time on children. J Paediatr Child Health.

[17] Zhao J, Zhang Y, Jiang F, Ip P, Ho FK, Zhang Y, Huang H (2018). Excessive screen time and psychosocial well-being: the mediating role of body mass index, sleep duration, and parent-child interaction. J Pediatr.

